# Supplementary material for: Specific phosphoinositide interaction of Jps1 is a key feature during unconventional secretion in Ustilago maydis
Source: J Biol Chem. 2025 May 8;301(6):110215. doi: 10.1016/j.jbc.2025.110215 (PMC12167484; doi:10.1016/j.jbc.2025.110215)
Supplement: Supplementary Material [file mmc1.pdf]

Supporting information

Specific phosphoinositide interaction of Jps1 is a key feature during unconventional secretion in *Ustilago maydis*

Sanchi Dali[1], Michèle Schultz[1], Marian Köster[1], Michael Kamel[2], Max Busch[2], Wieland Steinchen[3,4], Sebastian Hänsch[5], Athanasios Papadopolous[6], Jens Reiners[6], Sander H. J. Smits[6], Alexej Kedrov[2], Florian Altegoer[1#], Kerstin Schipper[1#]

[1] Institute of Microbiology, Heinrich Heine University Düsseldorf, Universitaetsstr. 1, 40225 Düsseldorf, Germany

[2] Synthetic Membrane Systems, Institute for Biochemistry, Heinrich Heine University Düsseldorf, Universitaetsstr. 1, 40225, Düsseldorf, Germany

[3] Department of Chemistry, Philipps University Marburg, Hans-Meerwein-Straße 4, 35043 Marburg, Germany

[4] Center for Synthetic Microbiology, Philipps University Marburg, Karl-von-Frisch-Straße 14, 35043 Marburg, Germany

[5] Center of Advanced Imaging (CAi), Heinrich Heine University Düsseldorf, Universitaetsstr. 1, 40225, Düsseldorf, Germany

[6] Center for Structural Studies, Heinrich Heine University Düsseldorf, Universitaetsstr. 1, 40225, Düsseldorf, Germany

[#]For correspondence: florian.altegoer@hhu.de; kerstin.schipper@hhu.de

Running title

Insights into the lipid interaction mechanism of Jps1

Keywords

cytokinesis, unconventional secretion, protein-lipid interaction, fungi, liposome, membrane binding

Table S1. Overall SAXS data from Jps1[1-484].

**Data collection parameters**	
SAXS Device	P12, PETRA III, DESY Hamburg (54)
Detector	PILATUS 6 M (423.6 x 434.6 mm^2)
Detector distance (m)	3.0
Beam size	120 µm x 200 µm
Wavelength (nm)	0.124
Sample environment	Quartz glass capillary, 1 mm ø
Absolute scaling method	Comparison with scattering from pure H_2O
Normalization	To transmitted intensity by beam-stop counter
Scattering intensity scale	Absolute scale, cm^{-1}
s range (nm^{-1})‡	0.03 – 4.4
**Sample**	
Organism	*Ustilago maydis* (strain 521 / FGSC 9021)
UniProt ID (range)	A0A0D1C3B2 (1-484)
Mode of measurement	batch
Temperature (°C)	10
Exposure time (# frames)	0.095 (40)
Protein buffer	20 mM HEPES pH 7.5, 20 mM KCl, 200 mM NaCl
Protein concentration (mg/ml)	0.6
**Structural parameters**	
Guinier Analysis (PRIMUS)	
$I(0) \pm \sigma$ (cm^{-1})	0.093 ± 0.00025
$R_g \pm \sigma$ (nm)	4.05 ± 0.02
s-range (nm^{-1})	0.066 – 0.320
min < sRg < max limit	0.268 – 1.294
Data point range	1 - 89
Linear fit assessment (R^2)	0.992
PDDF/P(r) Analysis (GNOM)	
$I(0) \pm \sigma$ (cm^{-1})	0.094 ± 0.00027
$R_g \pm \sigma$ (nm)	4.20 ± 0.02
D_{max} (nm)	14.65
Porod volume (nm^3)	253.61
s-range (nm^{-1})	0.066 – 4.087
χ^2 / CorMap P-value	0.950 / 0.746
**Molecular mass (kDa)**	
From *I*(0)	119.86
From Vc (64)	110.03
Bayesian Inference (65)	130.88
GNNOM (66)	131.10
From sequence	60.80 (monomer)<br>121.60 (dimer)
**Rigid body modeling**	
CORAL	
Symmetry	P1
s-range for fit (nm^{-1})	0.066 – 4.087
χ^2, CorMap *P*-value	1.06 / 0.0026
**SASBDB accession codes** (63)	
**Software**	
ATSAS Software Version (55)	3.0.5
Primary data reduction	SASFLOW / PRIMUS (57)
Data processing	GNOM (59)
Rigid body modelling	CORAL (60)
Model visualization	PyMOL (67)

‡$s = 4\pi \sin(\theta)/\lambda$, 2θ – scattering angle

Table S2. Oligonucleotides used in this study.

Number	Sequence (5´ to 3`)	Reference
oCD807	AGGAGGGTCTCCTCGAGAGCCGAACTACTCGGAGCATCCCA	This work
oEF319	GGTCTCCCATGGGCCCAGGCATCTCCAAGAAGCCTTCTT	This work
oEF320	GGTCTCCTCGAGATCGTCTTGCAGAGCGCCGG	This work
oEF333	GGTCTCCCATGGGCCCAGGCATCTCCAAGAAGCCCTC	This work
oEF334	GGTCTCCTCGAGGGATTCGGCGTCGATGGGCG	This work
oEF483	GGTCTCCTCGAGCCATCGAAAGATGCTCTTTTTTTTCTCC	This work
oEF517	GGTCTCCTCGAGCCAACGAAAAATGGACTTC	This work
oEF979	CCACGACTTGCAAATCGGATCCATGCCAGGCATCTCCAAGAAGCCTTC	This work
oEF980	CCTCGCCCTTGCTCACCATGAATTCAGCCGAACTACTCGGAGCATCCC	This work
oEF969	CCACGACTTGCAAATCGGATCCATGCCAGGCATCTCCAAGAAGCCCTCG	This work
oEF970	CCTCGCCCTTGCTCACCATGAATTCGGATTCGGCGTCGATGGGCGTTCG	This work
oEF987	GATAAGCTGTCAAACATGAGAATACATCGATGATATCAGATCTGC	This work
oEF988	GCAGATCTGATATCATCGATGTATTCTCATGTTTGACAGCTTATC	This work
oEF989	CCACGACTTGCAAATCGGATCCATGGACGATGGCCTGGTCCGCATG	This work
oEF990	CCTCGCCCTTGCTCACCATGAATTCCCAACGAAAAATGGACTTC	This work
oEF997	CCTCGCCCTTGCTCACCATGAATTCATCGTCTTGCAGAGCGCCGG	This work
oMB945	CATATGCCAGGCATCTCCAAGAAGCC	This work
oMB946	CTCGAGGGATTCCGCATCGATTGGGGTTTGG	This work
oRL1085	CACCATATGTTTGGACGTCTTAAGCACAGGATGTCTCGCGCTCGACTAGACG	(43)
oRL1086	GTGGGATCCTTACTTGAGGCCGTTCTTGACATTGTCCC	(43)
oXA21	GGTCTCCAAGCCAATGCGGCTGCGGCTTCGTTCGCTCCTTCTGGC	This work
oXA22	GGTCTCGGCTTGCGCCGCTGCCTCCACTGCTGCCGCCTGAGC	This work
oXA28	GGTCTCCGCCGAAGAAGAAAGCTGGACAGGAGTGGG	This work

oXA29	GGTCTCGCGGCCGCCGCCGCGACGACGGTGCCAGAAGGAGCG	This work
oXA241	GGTCTCCGGTGGCGCTGGCGGAGAACCCTCATGTCTCGG	This work
oXA242	GGTCTCCCACCGTCGCCGCGTGAGGCGAGACGTGAGGACTGCC	This work
oXA243	GGTCTCGTCGGCGATCTTTACCGGTGGTCATGGATACCCCGGTGTTCC	This work
oXA244	GGTCTCGCCGACGCAGGAGAGAGGAAAGCCGAG	This work
oXA245	GGTCTCGCTCGCGGCTCAGGCGGCAGCAGTGG	This work
oXA246	GGTCTCGCGAGCGCATCGCAGTTCATGATGCTCG	This work

Table S3. Plasmids used in this study. For detailed description see main text.

Plasmid (collection no.)	Usage	Reference
pET22b	Protein overexpression with N-terminal 6His-tag	Novagen
pEMGB1 (pIL17)	Protein overexpression with N-terminal GB1-tag	(42, 68)
pET15b_6xHis_Cts1 (pUMa1951)	Protein expression of 6xHis-Cts1-full-length	(43)
pET22b_Jps1_6xHis (pUMa3257)	Protein expression of 6xHis-Jps1 full-length	This work
pET15b_6xHis_Jps1_Gfp (pUMa4285)	Protein expression of 6xHis-Jps1Gfp full-length	This work
pPjps1_Jps1_eGfp_CbxR (pUMa3293)	Generation of plasmid pUX168 (via site directed mutagenesis)	This work
pEMGB1_Jps1_1-218 (pUX84)	Protein expression of GB1-Jps1_1-218	This work
pEMGB1_Jps1_1-484 (pUX85)	Protein expression of GB1-Jps1_1-484	This work
pEMGB1_SrJps1 (pUX91)	Protein expression of GB1-SrJps1 full-length	This work
pEMGB1_HcJps1 (pUX122)	Protein expression of GB1-HcJps1 full-length	This work
pEMGB1_Jps1$^{1\text{-}484}$M^{1} (pUX324)	Protein expression of GB1-Jps1M1	This work
pEMGB1_Jps1$^{1\text{-}484}$M^{2} (pUX325)	Protein expression of GB1-Jps1M2	This work
pEMGB1_Jps1$^{1\text{-}484}$M^{3} (pUX326)	Protein expression of GB1-Jps1M3	This work
pEMGB1_Jps1$^{1\text{-}484}$M^{4} (pUX185)	Protein expression of GB1-Jps1M4	This work
pEMGB1_Jps1$^{1\text{-}484}$M^{5} (pUX186)	Protein expression of GB1-Jps1M5	This work

pPjps1_eGfp_nosT_T2857A_CbxR (pUX168)	Backbone vector for generation of pUX169, pUX170, pUX171, pUx172, pUX178, pUX266	This work
pPjps1_SrJps1_eGfp_CbxR (pUX169)	Generation of complementation strain for SrJps1-eGfp (UX140)	This work
pPjps1_HcJps1_eGfp_CbxR (pUX171)	Generation of complementation strain for HcJps1-eGfp (UX141)	This work
pPjps1_Jps1_1-484_eGfp_CbxR (pUX172)	Generation of complementation strain for Jps11-484-eGfp (UX130)	This work
pPjps1_Jps1_1-218_eGfp_CbxR (pUX178)	Generation of complementation strain for Jps11-218-eGfp (UX134)	This work
pPjps1_Jps1$^{1-484}$M^{5}_eGfp_CbxR (pUX266)	Generation of complementation strain for Jps1M5-eGfp (UX207)	This work

Table S4. Strains used in this study.

Strain	Internal strain collection number	Parental strain	Vector	Genotype	Reference
U. maydis					
AB33	UMa133		-	*a2 Pnar: bW2bE1 PhleoR*	(46)
UM521	UMa54			*a1b1*	(69)
AB33 jps1Δ	UMa2092	UMa133		*a2 Pnar: bW2bE1 PhleoR umag_03776Δ_HygR*	(21)
AB33 Jps1G	UMa1847	UMa133	-	*a2 Pnar: bW2bE1 PhleoR umag03776- egfp_HygR*	(21)
AB33 jps1Δ/ Jps1$^{1-484}$G	Ux130	UMa2092	pUX172	*a2 Pnar: bW2bE1 PhleoR umag_03776Δ_HygR_ip^{r}(pJps1_Jps1_1-484_eGfp_nosT) ip^{s} CbxR*	This work
AB33 jps1Δ/ Jps1$^{1-218}$G	UX134	UMa2092	pUX178	*a2 Pnar: bW2bE1 PhleoR umag_03776Δ_HygR_ip^{r}(pJps1_Jps1_1-218_eGfp_nosT) ip^{s} CbxR*	This work
AB33 jps1Δ/ SrJps1G	Ux140	UMa2092	pUX169	*a2 Pnar: bW2bE1 PhleoR umag_03776Δ_HygR_ip^{r}(pJps1_SrJps1_eGfp_nosT) ip^{s} CbxR*	This work
AB33 jps1Δ/ HcJps1G	Ux141	UMa2092	pUX171	*a2 Pnar: bW2bE1 PhleoR umag_03776Δ_HygR_ip^{r}(pJps1_HcJps1_eGfp_nosT) ip^{s} CbxR*	This work

AB33 jps1Δ/ Jps1$^{1-484}$M^{5}G	UX207	UMa2092	pUX266	*a2 Pnar: bW2bE1 PhleoR umag_03776Δ_HygR_ip^{r}(pJps1_Jps1$^{1-484}$M^{5}_eGfp_nosT) ip^{s} CbxR*	This work
**Others**					
Sporisorium reilianum SRZ2	UMa695 (RK106)		-	*a2b2*	(44)

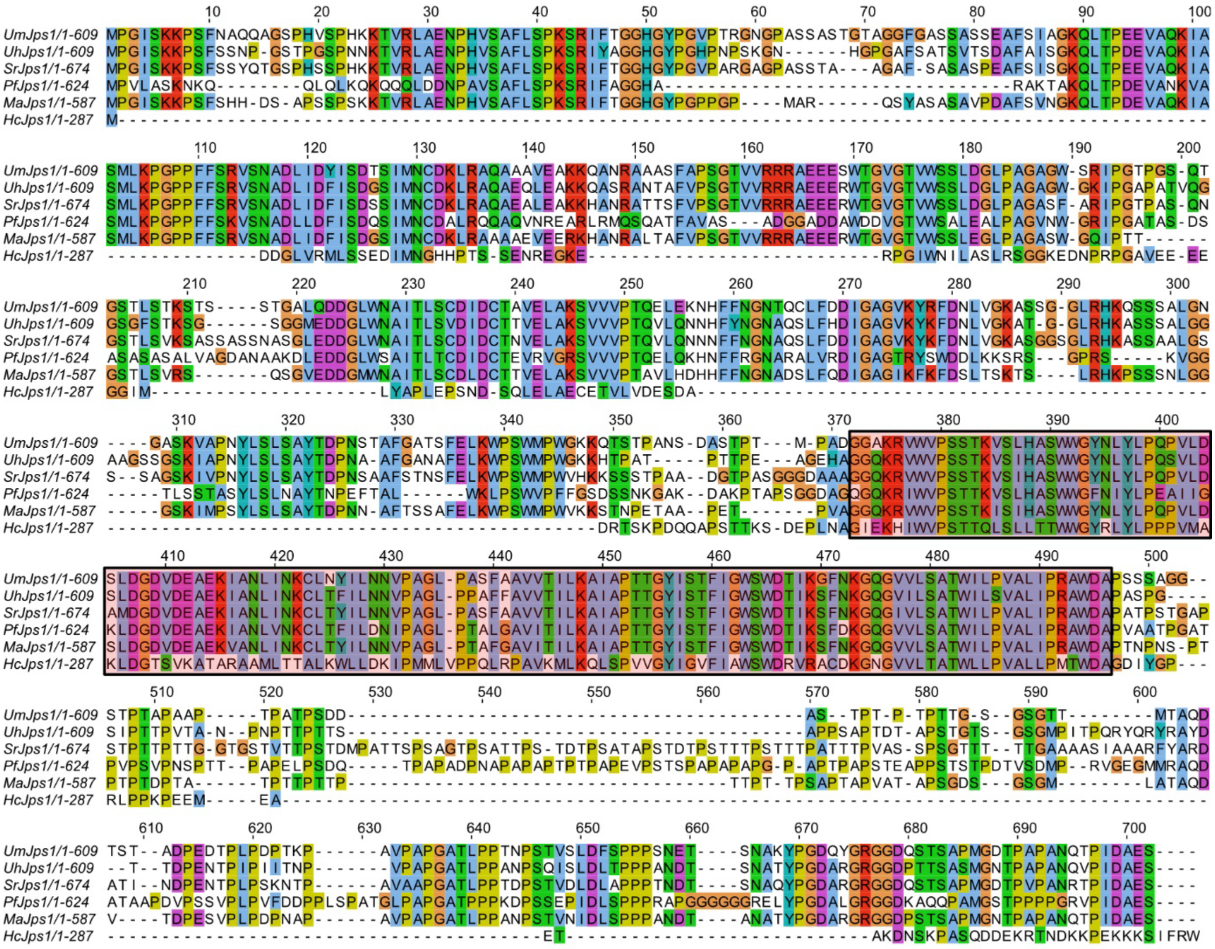

Supplementary Figure 1. Amino acid alignment of Jps1 and selected homologs. Homologous sequences were identified by BlastP and examples ranging from close relatives (smut fungi) to more distantly related Basidiomycetes were selected. The boxed region resembles the highly conserved core domain. The alignment was generated using the Clustal Omega webserver (70) and visualized using JalView (71). *Um, Ustilago maydis; Uh, Ustilago hordei; Sr, Sporisorium reilianum; Pf, Pseudozyma flocculosa; Ma,Moesziomyces antarcticus; Hc, Hebeloma cylindrosporium*. Numbers indicate protein lengths (amino acids).

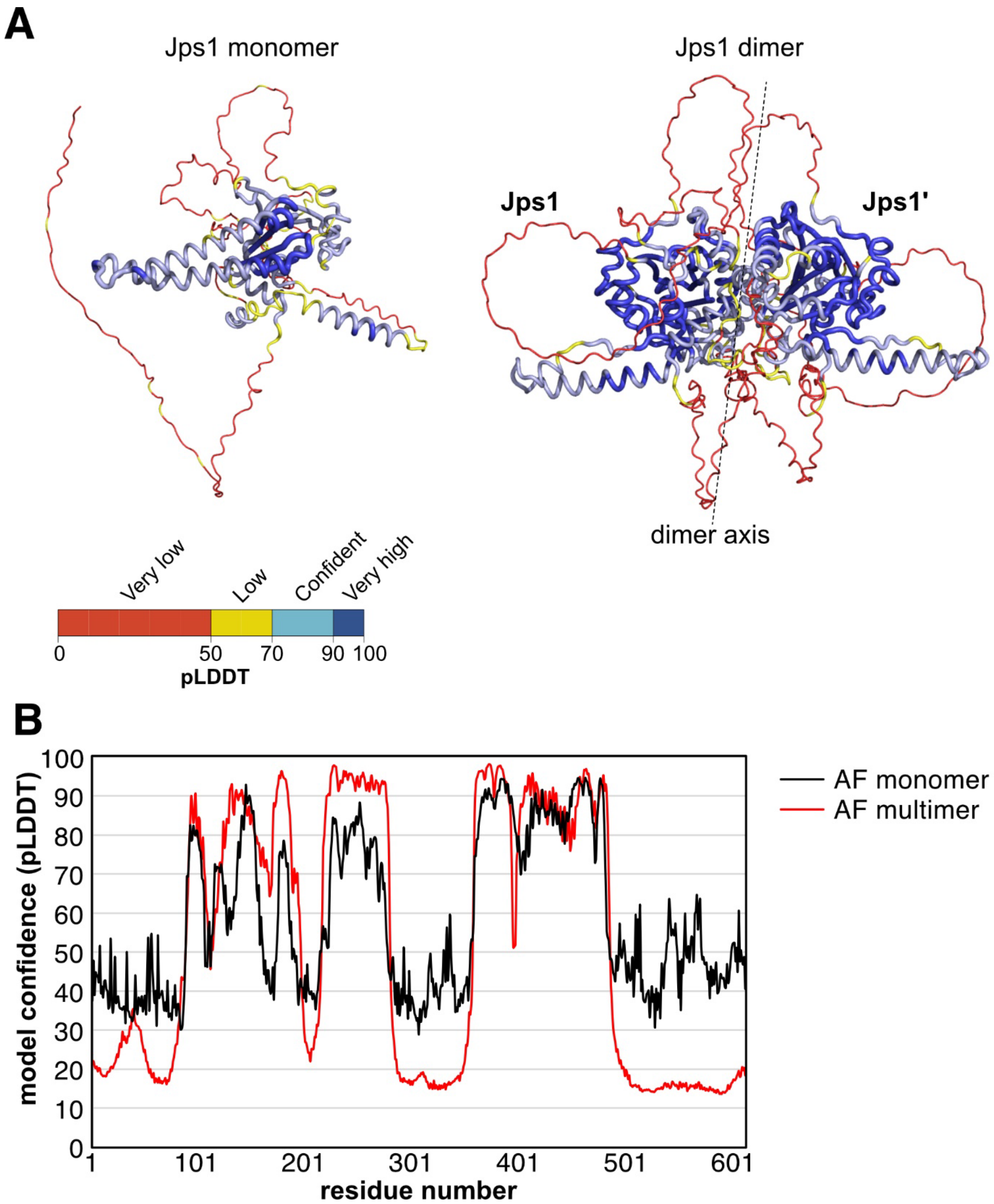

Supplementary Figure 2. Structure prediction of Jps1 using AlphaFold2. A. Structural models of the Jps1 monomer (left) and dimer (right) colored according to pLDDT values predicted with AlphaFold2 (24). **B.** Model confidence (pLDDT) score of a Jps1 monomer (black) and dimer (red). The pLDDT is plotted per residue. Notably, the pLDDT in the conserved regions improved from around 80 to >95, while it strongly dropped in the less conserved regions suggesting intrinsically disordered regions.

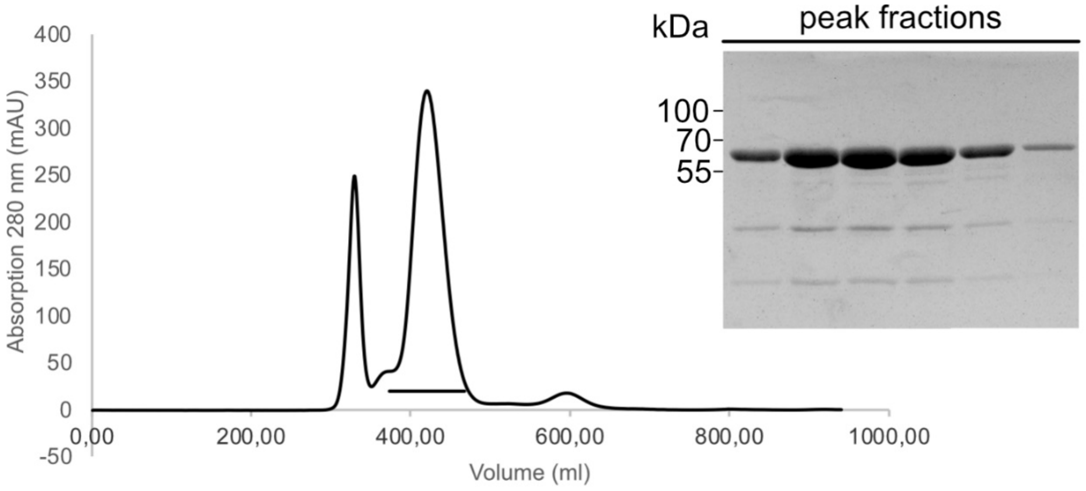

Supplementary Figure 3. Purification of Jps1. Shown are a chromatogram of full-length Jps1 from the size-exclusion chromatography and an SDS-PAGE of the peak fractions marked with a black line.

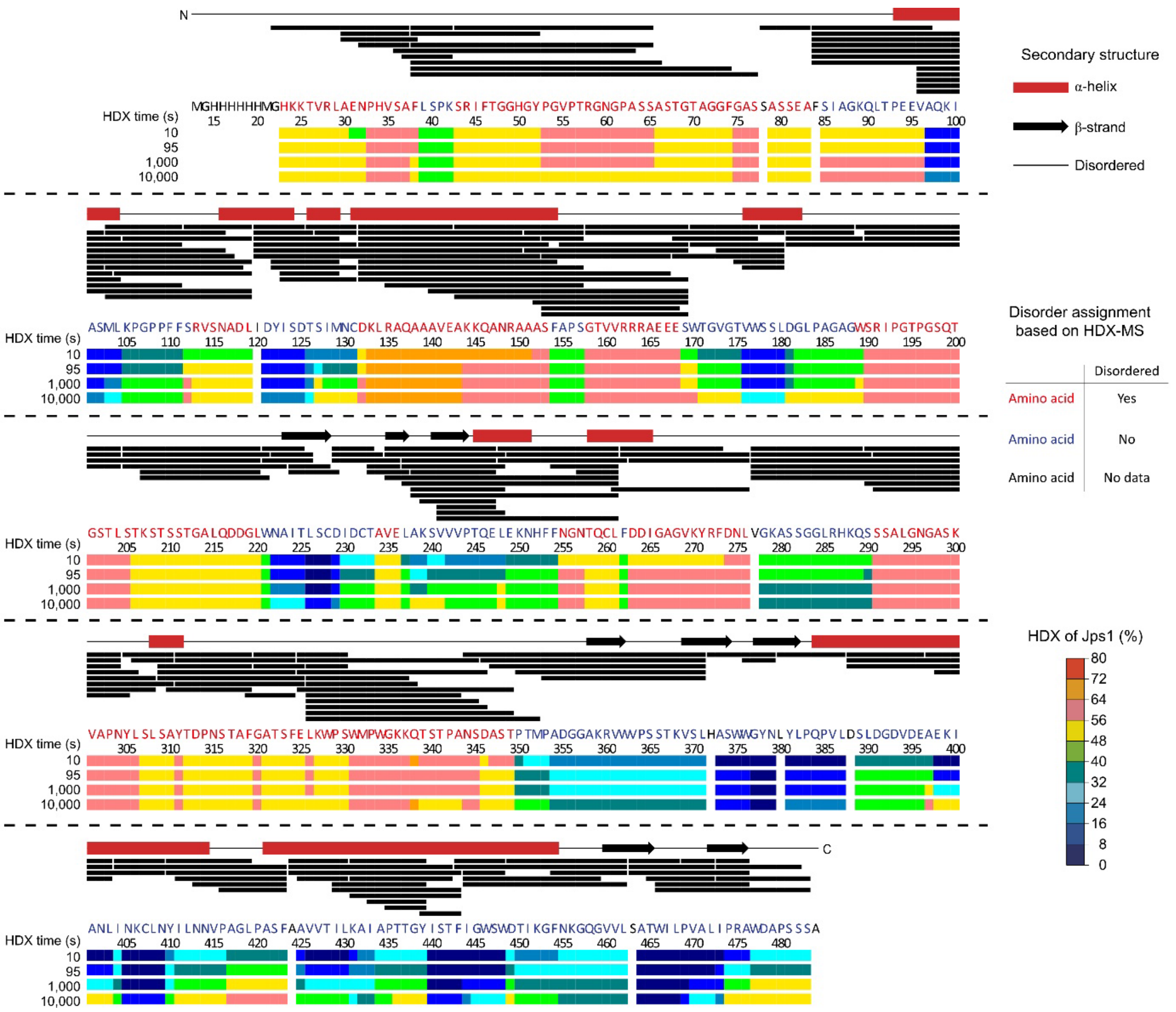

Supplementary Figure 4. Disordered regions of Jps1 in solution probed by HDX-MS. The residue-specific HDX of Jps1 is plotted on its amino acid sequence. Each black bar represents a peptide identified in HDX. The secondary structure as per the AlphaFold2 model of Jps1 (21) is illustrated above. Residues are colored in red or blue for residues predicted as disordered or possessing higher-order structure respectively, and residues not covered by peptides are colored black.

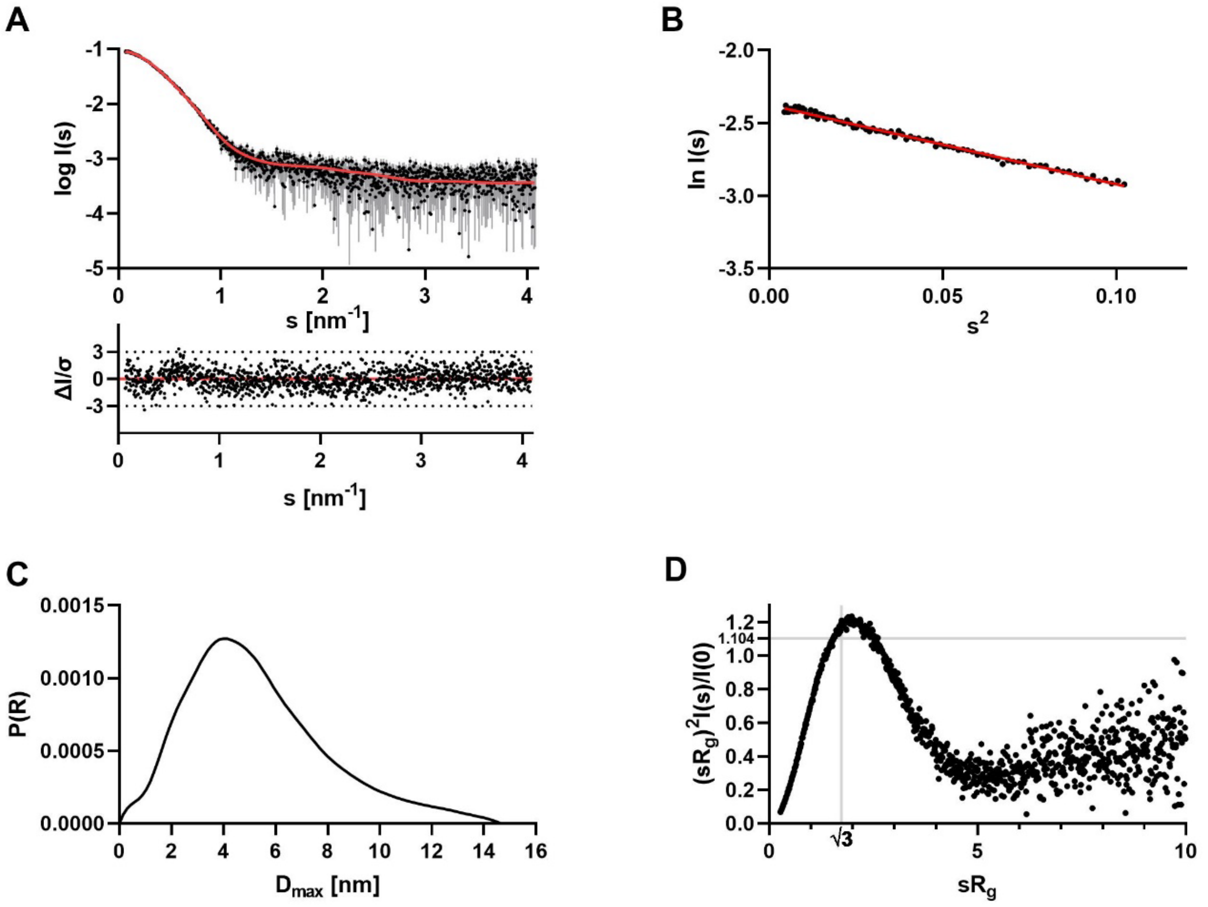

Supplementary Figure 5. Small-angle X-ray scattering data from Jps1$^{1\text{-}484}$. A. Scattering data of Jps1$^{1\text{-}484}$. Experimental data are shown in black dots, with grey error bars. The CORAL model fit is visualized as red line and below is the residual plot of the data. **B.** The Guinier plot of Jps1$^{1\text{-}484}$ showed a stable Guinier region with a R_g of 4.05 nm. **C.** The *p(r)* function of Jps1$^{1\text{-}484}$ shows a maximum intra-particle distance (D_{max}) of 14.65 nm. **D.** The dimensionless Kratky plot of Jps1$^{1\text{-}484}$ revealed a compact elongated molecule.

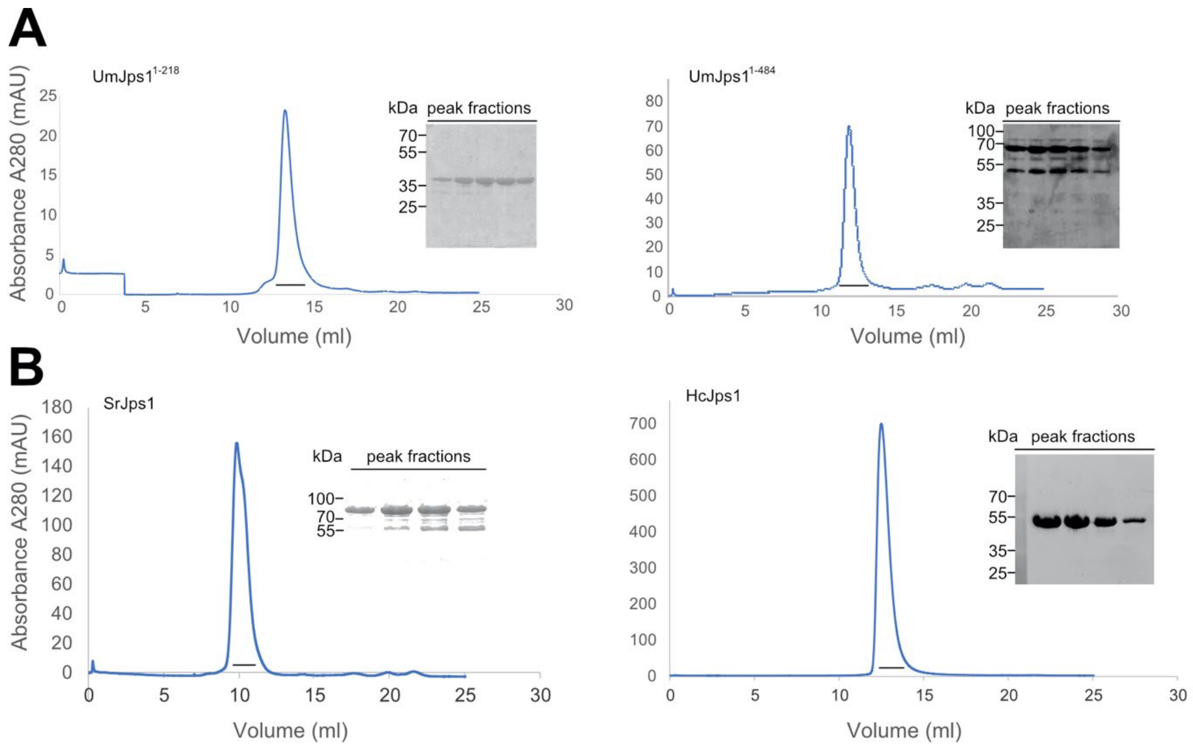

Supplementary Figure 6. Purification of Jps1 truncations and homologs. A. Expression of UmJps1$^{1-218}$ and UmJps1$^{1-484}$ in *E. coli*. Shown are the chromatograms obtained from size-exclusion chromatography. The Coomassie-stained SDS-PAGE show peak fractions as indicated in the chromatogram. **B.** Expression of full-length SrJps1 and HcJps1 in *E. coli*. Shown are the chromatograms obtained from size-exclusion chromatography. The Coomassie-stained SDS-PAGE show peak fractions as indicated in the chromatogram.

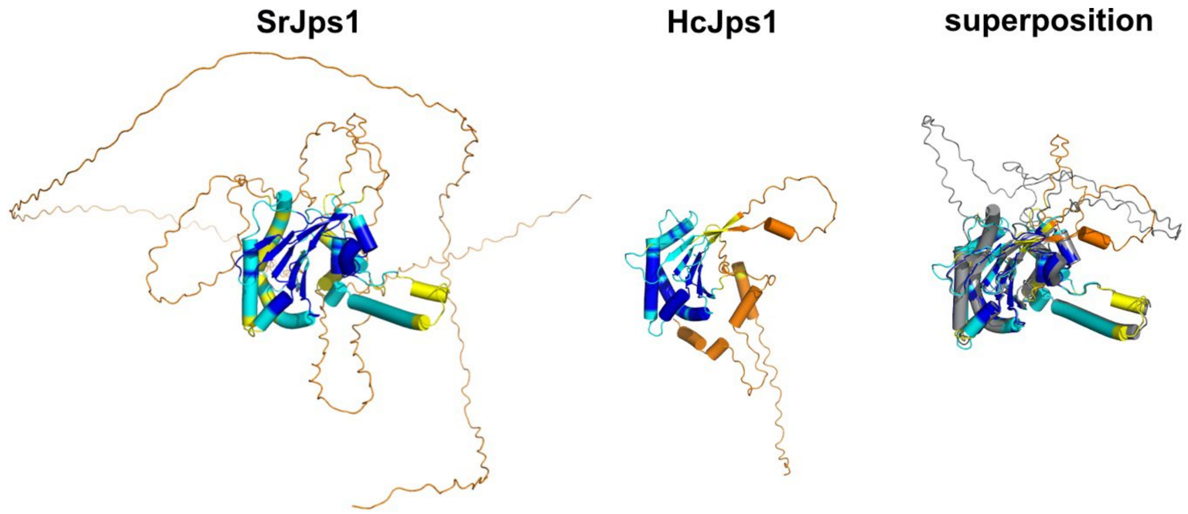

Supplementary Figure 7. Structure prediction of SrJps1 and HcJps1. Shown are the AlphaFold2 structural models of SrJps1 and HcJps1 colored by confidence. Structural models were superposed to UmJps1 and the flexible N- and C-termini removed to visualize that the conserved core domains align well.

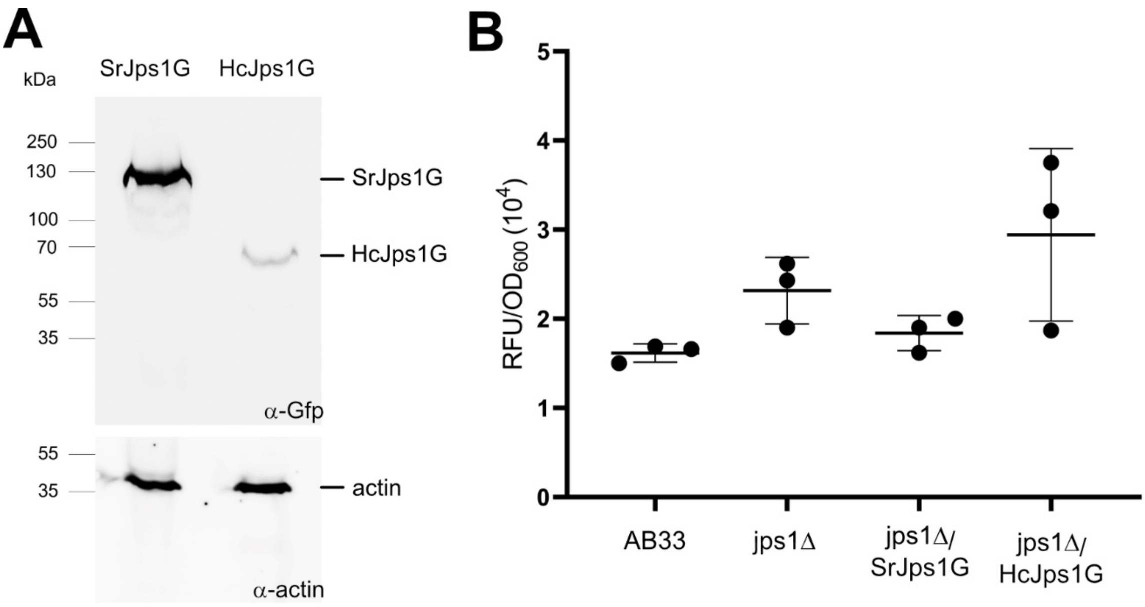

Supplementary Figure 8. Intracellular Cts1 activity of indicated AB33 derivatives. A. Western blots of SrJps1G and HcJps1G fusion proteins detected in cell extracts. Actin was used as loading control. **B.** Scatter plots depicting intracellular chitinase activity. Background strain AB33 and AB33 jps1Δ lacking Jps1 were used as controls. Intracellular chitinase activity of AB33 jps1Δ/SrJps1G was similar to AB33 while activity of AB33 jps1Δ/HcJps1G was elevated resembling loss of Jps1. The assay was conducted in three biological replicates. Error bars indicate standard deviation.

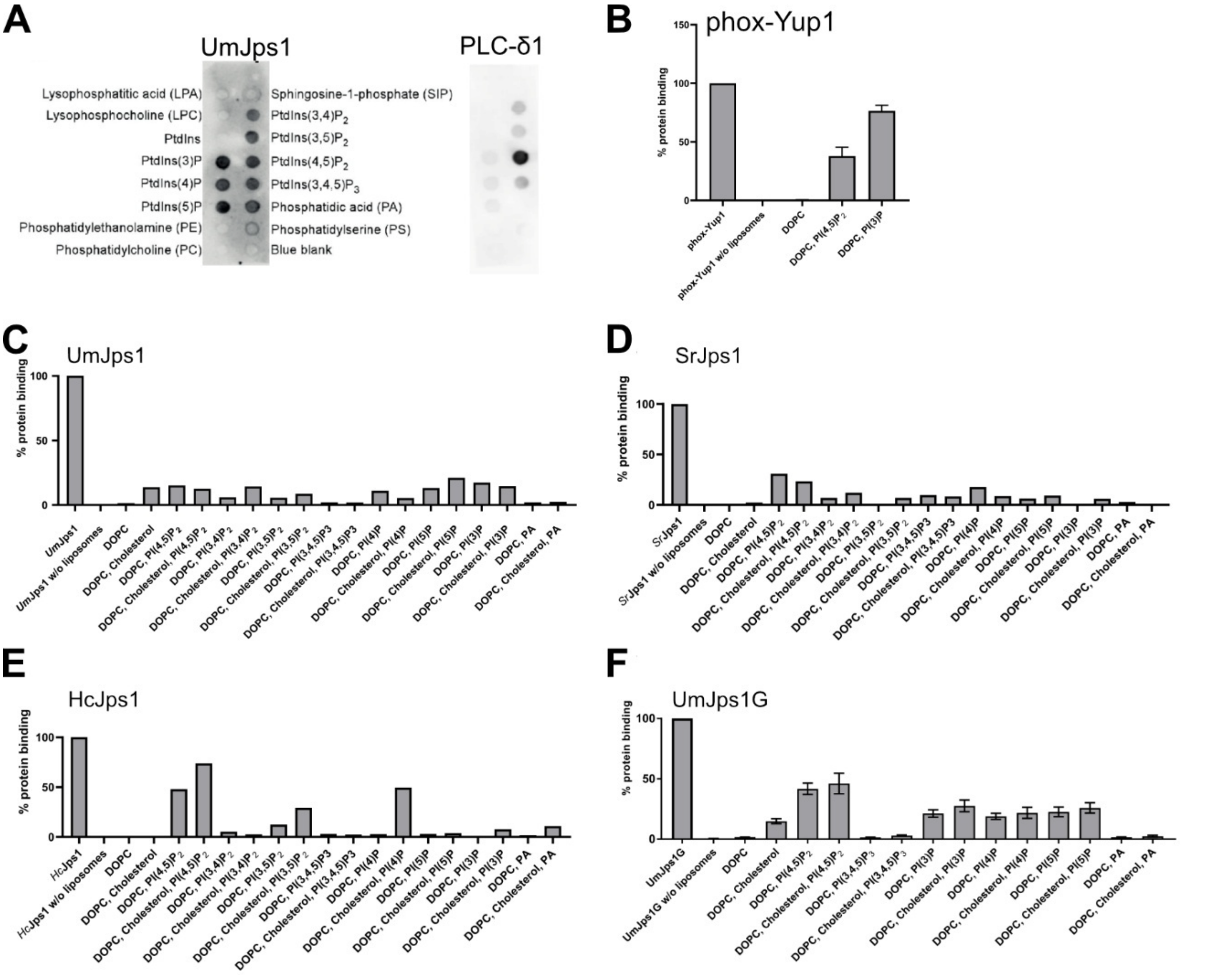

Supplementary Figure 9. Lipid-protein interaction assays using PIP strips and liposome binding assays. A. PIP strips were incubated with recombinant His-tagged Jps1. Antibodies directed against the His-tag were used for detection. Recombinant PLC-δ1 PH domain protein (Echelon) specifically binding PI(4,5)P_2 was employed as positive control. **B. - E.** Quantification of liposome binding assays using the PX domain of UmYup1 (phox-Yup1). (**B**), UmJps1 (**C**), SrJps1 (**D**), HcJps1 (**E**) and UmJps1G (**F**). Liposomes containing DOPC plus the indicated lipids were incubated with recombinant Jps1 variants. Interacting proteins localize to the pellet fraction after high-speed centrifugation. Liposome binding assays were conducted once for screening purposes (**C.-E.**), the data shown in **B** and **F** represent the mean of biological triplicates. Error bars represent standard deviation.

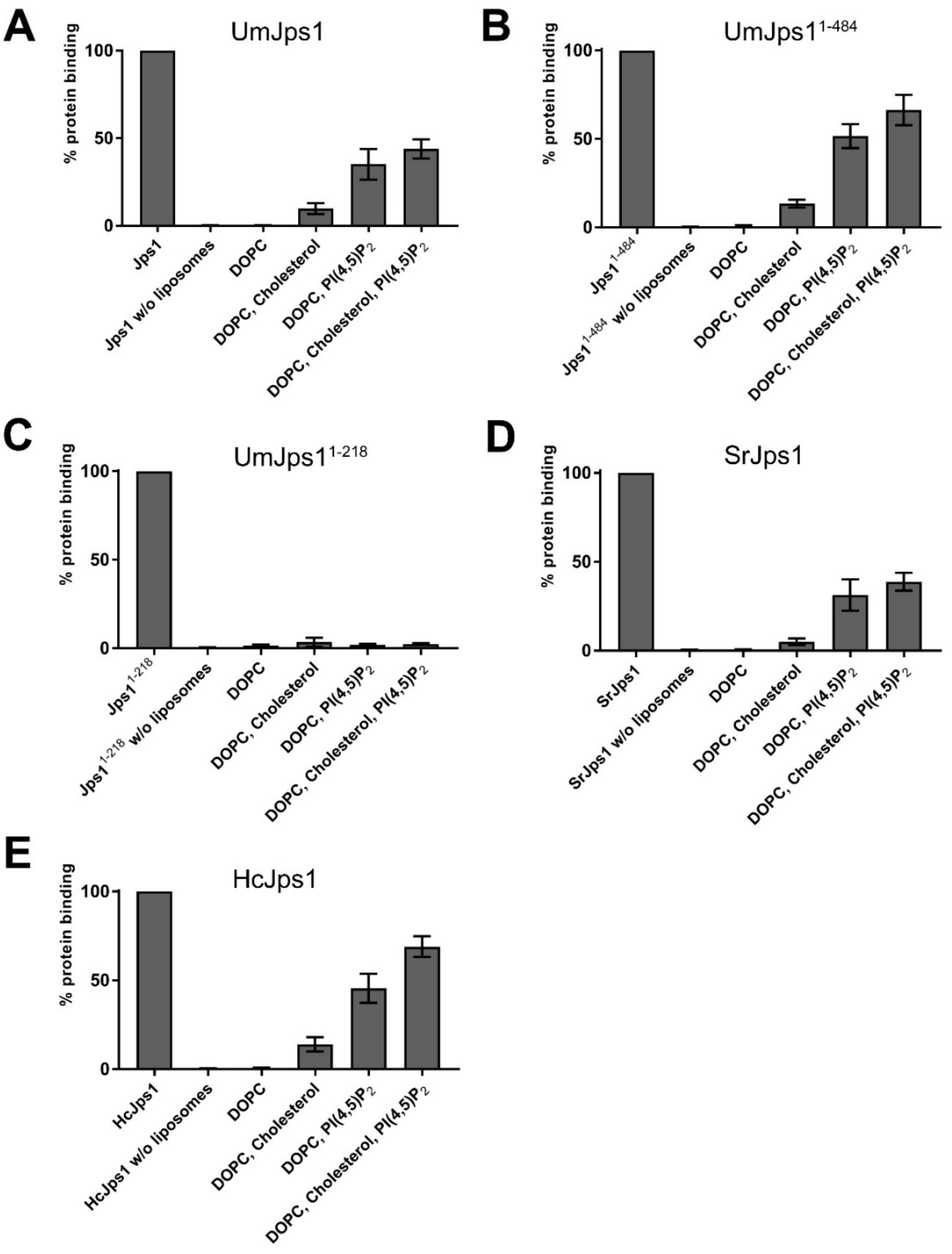

Supplementary Figure 10. Liposome-binding assays to corroborate PI(4,5)P_2 interaction. Quantification of pellet fraction from liposome binding assays using the purified proteins UmJps1 (**A**), UmJps1$^{1-484}$ (**B**), UmJps1$^{1-218}$ (**C**), SrJps1 (**D**), HcJps1 (**E**) and incubated with the indicated lipids and lipid mixtures. All assays were conducted in biological triplicates. Error bars represent standard deviation.

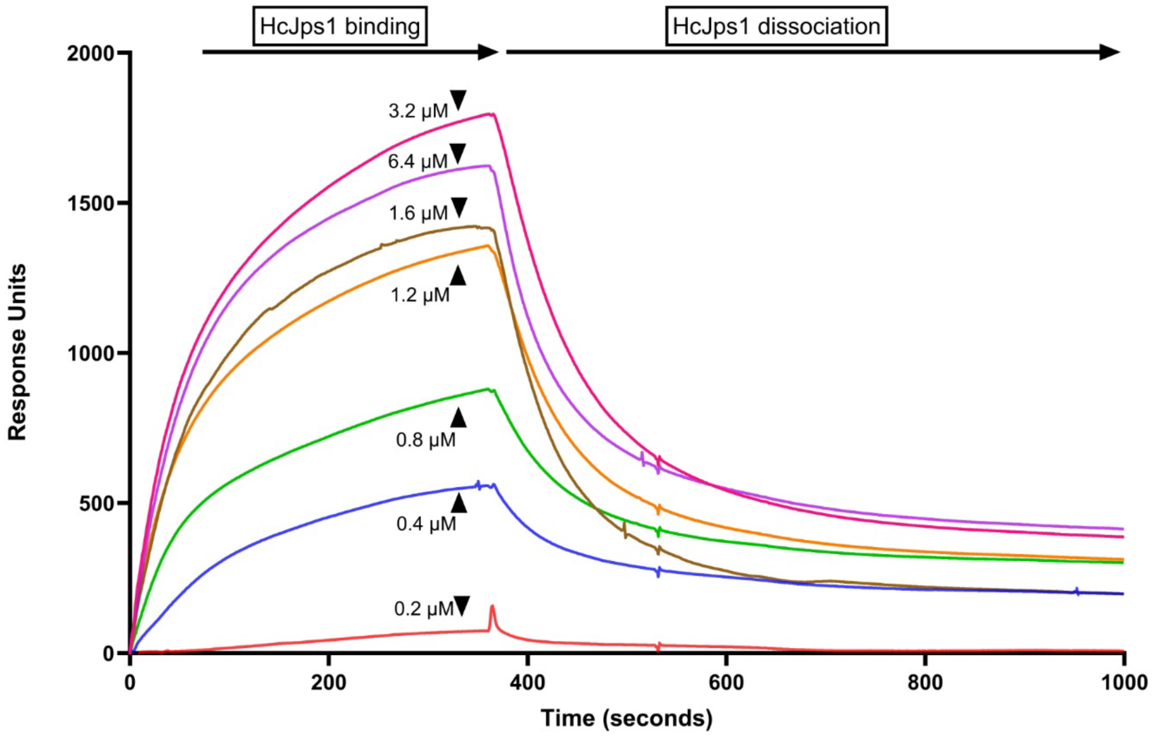

Supplementary Figure 11. Surface Plasmon Resonance (SPR) of HcJps1. Shown here is the concentration range of HcJps1 at 0.2 µM to 6.4 µM binding to the immobilized liposomes containing DOPC and PI(4,5)P_2, followed by the dissociation phase. DOPC-only liposomes were used as a negative control in the reference channel. The response units of a reference buffer (blank) was subtracted from all depicted curves.

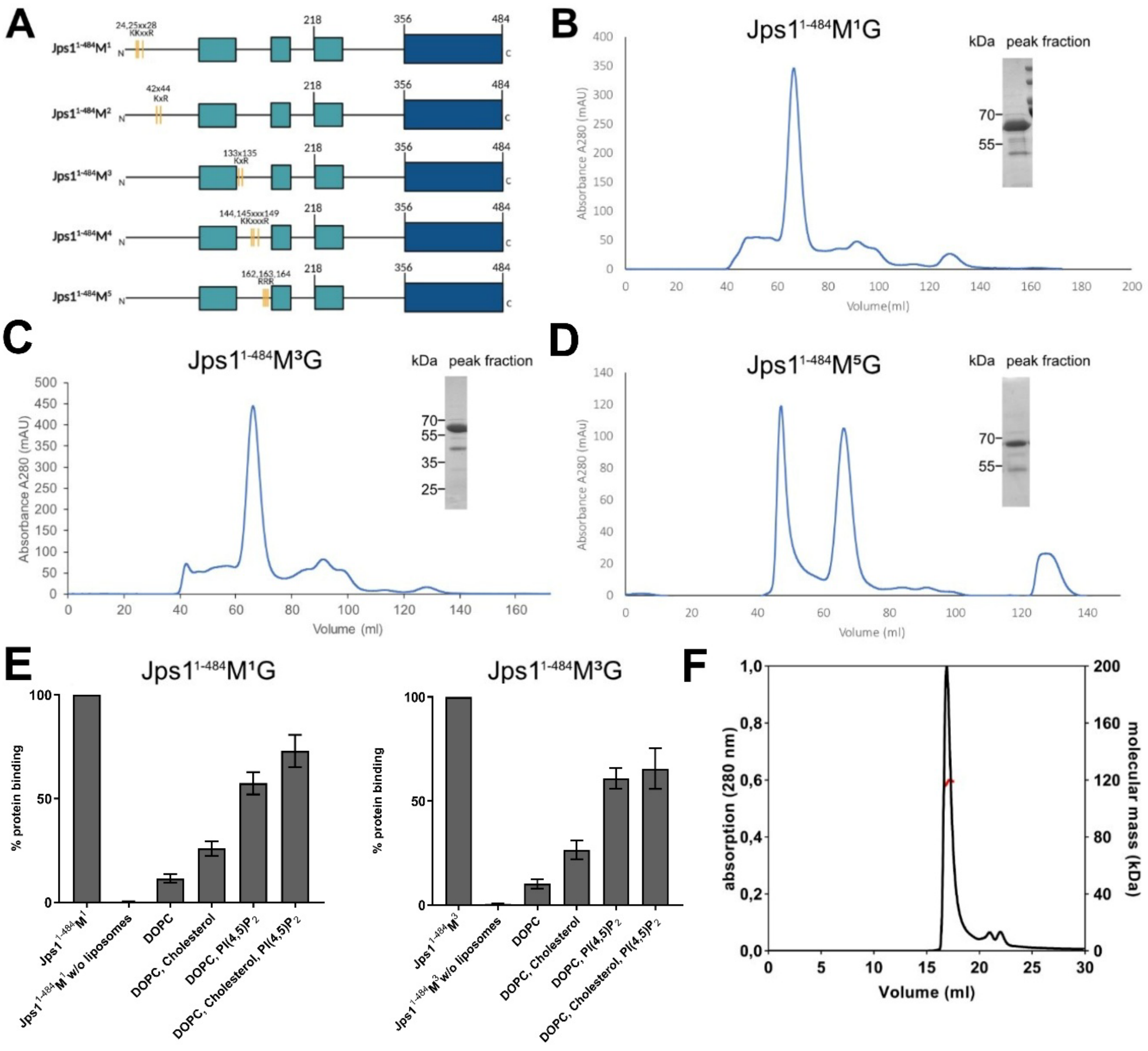

Supplementary Figure 12. Purification and lipid-interaction assays of Jps1$^{1\text{-}484}$ variants with mutated basic clusters. A. Schematic representation of the different Jps1 mutant versions. Mutated residues are indicated in light orange. Basic amino acids (Lysin, Arginine) were replaced by Alanine. **B-D.** Expression of UmJps1$^{1\text{-}484}$M^1, -M^3 and -M^5 in *E. coli*. Shown are the chromatograms obtained from size-exclusion chromatography. The Coomassie-stained SDS-PAGE show peak fractions as indicated in the chromatogram. **E.** Quantification of liposome binding assays. Liposomes containing DOPC plus the indicated lipids were incubated with recombinant Jps1$^{1\text{-}484}$ variants M^1 and M^3. Interacted proteins localize to the pellet fraction after high-speed centrifugation. The assay was performed in biological triplicates. Error bars depict standard deviation. **F.** SEC-MALS anaylsis of Jps1$^{1\text{-}484}$M^5. The black line shows the absorption at 280 nm (SEC), the molecular weight as determined by MALS is depicted in red.

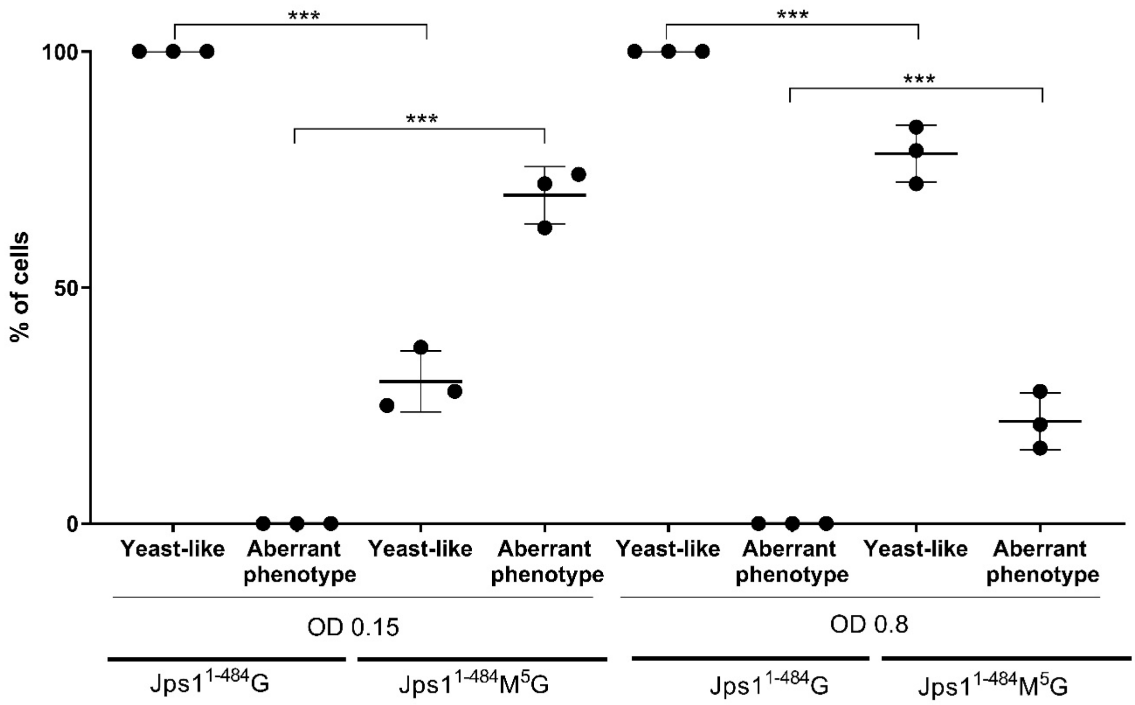

Supplementary Figure 13. Statistic evaluation of morphological disturbances. Scatter plot depicting quantification of yeast-like and aberrant morphology in cells of Jps1$^{1-484}$G and Jps1$^{1-484}$M^{5}G at both low (0.15) and normal (0.8) optical density (OD_{600}). n=1000, performed in three biological replicates. Error bars indicate standard deviation. ***P-value 0.001 (two sample t-test).

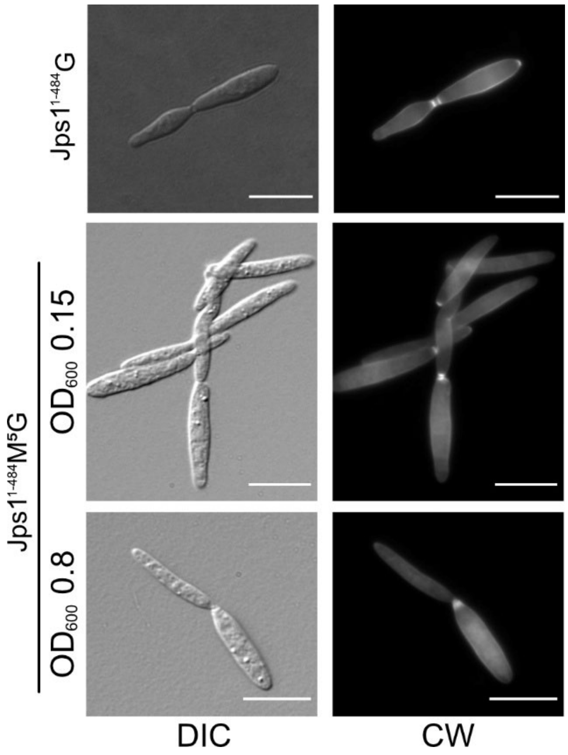

Supplementary Figure 14. Morphology and septal staining in strains expressing Jps1$^{1-484}$ and its variant M^5. Septal staining using Calcofluor White of the AB33 jps1Δ complementation strains Jps1$^{1-484}$G, and Jps1$^{1-484}$M^{5}G at low and normal optical density (OD$_{600}$). DIC, differential interference contrast. CW, Calcofluor White. Scale bars, 10 µm.
